# 1113. Case Study. Conservative Enzymatic Debridement for Full-thickness Eschar Removal in a Low-hemoglobin Jehovah’s Witness Patient Refusing Transfusion

**DOI:** 10.1093/jbcr/irag033.287

**Published:** 2026-04-06

**Authors:** Michelle K Hughes, Annie Cate Schmidt, Anne Seyferth, William Hughes

**Affiliations:** Jefferson Burn Center, Thomas Jefferson University Hospital, Philadelphia, PA; Sidney Kimmel Medical College, Philadelphia, PA; Sidney Kimmel Medical College, Philadelphia, PA; Jefferson Burn Center, Thomas Jefferson University Hospital, Philadelphia, PA

## Abstract

**Patient Presentation (age range, injury details, relevant history):**

A 51-year-old female presented with a 45% TBSA burn injury, predominantly full thickness, and associated inhalation injury from a structure fire. The patient was taken to the OR within 24 hours for removal of the full thickness burn from the anterior torso with placement of a dermal template and removal of the left upper extremity burn injury with placement of a polymer dressing. At that time, the starting hemoglobin of 13.2 g/dL decreased to 10.5 g/dL. Further excision and grafting decreased the hemoglobin to 6.8 g/dL. On post-burn day (PBD) 11, significant eschar remained on the bilateral anterior thighs and right arm.

**Clinical Challenges:**

The early removal of a full thickness burn injury is paramount in the treatment of the burned patient, with a demonstrated decrease in morbidity and mortality since this practice was initiated. After initial debridement, the patient’s hemoglobin decreased to below the transfusion threshold (typically below 7 g/dL). The patient required additional excision and grafting of their injuries; however, the patient identified as a Jehovah’s Witness and refused all blood transfusions.

**Management Approach:**

After the decrease in hemoglobin to 6.8 g/dL, enzymatic debridement, as opposed to surgical debridement, was performed to minimize blood loss and remove eschar before grafting. Further grafting over the dermal template decreased the hemoglobin to 4.9 g/dL. The patient was treated with IV iron and Epogen, which increased hemoglobin levels to above 7 g/dL, at which point grafting on the thighs and right arm was performed.

**Outcomes:**

Enzymatic debridement was effective in removing eschar and minimizing blood loss. The patient’s hemoglobin levels remained above the transfusion threshold and grafting was successfully performed.

**Lessons Learned:**

In this case of a Jehovah’s Witness patient refusing blood products, enzymatic debridement was effective in removing eschar and minimizing blood loss. Removing eschar has been shown to decrease the morbidity and mortality caused by burn wound infections and sepsis. The utilization of an enzymatic debridement agent can be beneficial in removing full thickness eschar in patients to minimize blood loss before grafting when blood transfusion is not an option.

**Applicability to Practice:**

This case demonstrates the use of enzymatic debridement as an alternative to surgical debridement for reducing blood loss during burn excision in patients who cannot receive blood transfusions, such as Jehovah’s Witnesses. It is a practical strategy that can inform the management of similar patients and contribute to the evidence for minimizing blood loss in burn care.

